# Incidence and risk factors of in-stent restenosis after percutaneous coronary intervention in patients from southern China

**DOI:** 10.1186/s40001-022-00640-z

**Published:** 2022-01-22

**Authors:** Mingrui Li, Jingyuan Hou, Xiaodong Gu, Ruiqiang Weng, Zhixiong Zhong, Sudong Liu

**Affiliations:** 1 Faculty of Graduate Studies, Guangdong Medical University, Zhanjiang, 524000 People’s Republic of China; 2 Center for Cardiovascular Diseases, Meizhou People’s Hospital (Huangtang Hospital), Meizhou, 514031 People’s Republic of China; 3 Research Experimental Center, Meizhou People’s Hospital (Huangtang Hospital), Meizhou, 514031 People’s Republic of China; 4 Guangdong Provincial Key Laboratory of Precision Medicine and Clinical Translational Research of Hakka Population, Meizhou, 514031 People’s Republic of China

**Keywords:** In-stent restenosis (ISR), Percutaneous coronary intervention (PCI), Drug-eluting stent (DES), Risk factor

## Abstract

**Background:**

In-stent restenosis (ISR) remains a challenge for coronary artery disease (CAD) patients who undergo percutaneous coronary intervention (PCI) with stents, and risk factors for ISR are controversial. This study aimed to investigate the incidence and risk factors of ISR in patients from southern China.

**Methods:**

In this retrospective study, patients diagnosed as acute coronary syndromes (ACS) and underwent successful PCI with drug-eluting stent (DES) and conducted a follow-up coronary angiography in Center for Cardiovascular Diseases of Meizhou People’s Hospital at the period of January 1st, 2016 to January 1st, 2021 were included for analysis. The clinical and angiographic factors were compared between patients in ISR ( +) and ISR (−) groups. The association between variables and ISR was evaluated by multivariate logistic regression model.

**Result:**

A total of 341 ACS patients who had been installed at least 1 stent were included in this study. The follow-up time was 34.2 ± 17.2 months. During the follow-up period, 62 (18.2%) patients experienced ISR, and the average time for ISR was 32.8 months; the incidence of ISR for left main coronary artery, left anterior descending coronary artery, left circumflex artery coronary artery and right coronary artery were 6.7%, 20.9%, 19.4% and 14.4%, respectively; left ventricular ejection fraction (LVEF), stent number, stent type, statin therapy, antiplatelet therapy were significantly different between patients in ISR ( +) and ISR (−) group. Multivariate logistic analysis suggested that LVEF and stent number were significantly correlated with ISR.

**Conclusion:**

Our study revealed the incidence and risk factors of ISR in patients from southern China. Our data suggested that LVEF and stent number were independent risk factors associated with ISR.

## Background

Coronary artery disease (CAD), whose pathological basic is the formation of atherosclerotic plaques/lesions in coronary arteries, claims more than 9 million lives globally and is the most deadly disease in the world [1]. Currently, percutaneous coronary intervention (PCI) with stent is a major therapeutic strategy for severe CAD cases, especially for those with acute coronary syndromes (ACS). PCI efficiently improves myocardial ischemia and protects against adverse vascular events in ACS patients [2]. Stent implantation is the main PCI and has great advantages over balloon angioplasty [3]. It is estimated that approximate 60% of ACS cases are treated with PCI, and this number is increasing annually [4, 5]. However, although interventional approaches and pharmacological therapies have advanced promptly, in-stent restenosis (ISR) remains a major challenge. ISR refers to > 50% stenosis inside or neighboring a previously stented segment, and is related to significant morbidity in patients after stent implantation [6, 7]. Studies suggested that ISR may cause the recurrence of major adverse cardiovascular events (MACEs) such as angina pectoris, acute myocardial infarction [8]. In the bare-metal stent (BMS) era, the clinical incidence of ISR was approximately 20–35%. Although the application of drug-eluting stents (DES) has significantly reduced the risk of ISR, ISR still happens in 5–10% of patients [9]. Clinically, there are some therapeutic strategies for ISR, including plain balloon angioplasty, DES implantation, drug-coated balloons angioplasty, rotational atherectomy and laser techniques, and so on [10]. However, there are lack of evidences from large-scale randomized trials to determine which option is the gold standard for ISR. To date, treatment of ISR remains a challenge and the optimum percutaneous treatment strategy is still debated [11]. Therefore, ISR still remains a major clinical issues after PCI and serves as an independent predictor for mortality post-PCI [12, 13].

Although evidences suggest that neointimal hyperplasia play an essential role in the development of ISR, the underlying etiology is yet to fully elucidate [14]. The use of intravascular imaging, i.e. intravascular ultrasound (IVUS) and optical coherence tomography (OCT), has allowed for better characterization of ISR, but these evaluation are invasive and expensive [15]. Identifying the risk factors of ISR is critical for predicting and preventing its occurrence in advance [11]. Kastrati et al. found that complex lesions (B2/C), restenosis, vessel size < 3 mm, stented segment > 15 mm and stent type are associated with ISR [16]. Several studies have suggested that diabetes mellitus, length (stent length) and small vessel size are the most predictive factors for ISR [17]. Lately, researchers found that patients who developed lesions in multiple vessels were vulnerable to ISR [18]. The level of C-reactive protein is a novel biomarker of ISR supported by several evidences [19]. Additionally, genetic polymorphism may exert a function in the progress of ISR, as studies showed that some SNPs in *CD18* and *MMP13* genes increase the risk of ISR [20, 21]. Dario et al*.* put forward that risk factors contributing to ISR could be divided into three divisions, which were patient-related, lesion-related and procedural-related [7]. However, most of these risk factors were evaluated for BMS implantation and evidence for DES implantation is limited, which highlights the necessity for studies in field.

In this study, we compared the patient-related, lesion-related and procedural-related features between CAD patients with ISR and without ISR from southern China. The aim of this study is to identify the risk factors of ISR, and provide practical information for the prevention of it.

## Methods

### Study subjects

This was a retrospective study. The protocol of the study was approved by the Ethical Committee of Meizhou People’s Hospital (Huangtang Hospital) (MPH-HEC 2021-C-31). The participants were diagnosed with ACS in Cardiology Department of Meizhou People’s Hospital during January 1st, 2016 to January 1st, 2019, and underwent successful PCI with DES implantation for the first time. Patients would go on for follow-ups after discharge from hospital. During follow-ups, patients presented indicated symptoms such as chest pain, shortness of breath were checked by cardiologists and a second coronary angiography was suggested. ISR was defined if the diameter of stenosis > 50% inside or neighboring the stent (< 5 mm). Patients were excluded from the study if they had following conditions (1) severe infectious diseases or malignant tumors; (2) follow-up coronary angiography data were missing. All these data were collected from our hospital electronic medical records system (EMR).

### Percutaneous coronary intervention and quantitative coronary angiography

PCI was performed using 6 Fr or larger guide catheter through radial or femoral approach to implant the DES. The PCI operations were performed by experienced interventional physicians. After PCI, patients received antiplatelet therapy (aspirin, clopidogrel or ticagrelor) for at least 1 year. QCA was performed using Innova IGS 530 (GE Co., France). Coronary angiograms were analyzed by experienced cardiologists. For follow-up angiography, QCA examined the stenosis in both in-stent and neighboring stent (< 5 mm).

### Clinical characteristics and laboratory parameters

The clinical characteristics of study subjects were collected from patients’ medical records. Laboratory parameters, such as total cholesterol (TC), triglyceride (TG), LDL-C, HDL-C, left ventricular ejection fraction (LVEF), C-reactive protein, cTnI and HbA1c examined before the PCI were collected for analysis. Hypertension was defined either presenting systolic blood pressure (SBP) > 140 mmHg or diastolic blood pressure (DBP) > 90 mmHg. Diabetes mellitus was diagnosed if fasting blood glucose ≥ 126 mg/dl or taking anti-diabetic treatment [22]. Dyslipidaemia was diagnosed if serum TC level of > 5.17 mmol/L, LDL-C level of > 4.14 mmol/L, TG level of > 1.7 mmol/L, HDL-C level of < 1.04 mmol/L or current treatment with anti-dyslipidemic medication.

### Statistical analysis

Statistical analyses were performed using SPSS version 18.0 (SPSS, Chicago, Illinois, USA). Data were presented as mean ± SD. Continuous variables were tested by Student’s t test, whereas categorical variables were analyzed by chi-square (χ^2^) test or Fisher’s exact test. Logistic multivariate regression analysis was used to evaluate the association between risk factors and ISR. Odds ratios (ORs) and 95% confidence intervals (CIs) were calculated by adjusting for variables such as gender, age, hypertension, diabetes mellitus, and stent diameter. A *P* value of < 0.05 was considered statistically significant.

## Results

### Baseline characteristics of study subjects

A total of 341 ACS patients (mean age 65.8 ± 10.9, 63% men) were included for analysis in the present study. The patients were divided into two groups (ISR [ +], ISR [−]) according to the presence of ISR during the follow-up. 62 of 341 (18.2%) patients had ISR. The baseline characteristics of the study subjects are presented in Table 1. The mean follow-up time is 34.2 ± 17.2 months. The mean implanted stent number is 1.34 ± 0.65. The ISR incidence is 6.7% in left main coronary artery (LM), 20.9% in left anterior descending coronary artery (LAD), 19.4% in left circumflex artery coronary artery (LCX), and 14.4% in right coronary artery (RCA), suggesting that LAD and LCX were more susceptible to ISR.

**Table 1:** Baseline characteristics of the ACS patients

Variables	patients (*n* = 341)
Age (years)	65.8 ± 10.9
Male, *n* (%)	253 (63.0)
Smoking, *n* (%)	67 (19.6)
Drinking, *n* (%)	6 (1.8)
Comorbidity	
Hypertension, *n* (%)	215 (63.0)
Diabetes mellitus, *n* (%)	137 (40.1)
Dyslipidemia, *n* (%)	82 (24.0)
Angiographic follow-up (months)	34.2 ± 17.2
Stent number (mm)	1.34 ± 0.65
Stent type	
Rapamycin, *n* (%)	275 (80.6)
Everolimus, *n* (%)	24 (7.0)
Zotamos, *n* (%)	28 (8.2)
Stent location	
LM, *n* (%)	15 (3.3)
LAD, *n* (%)	191 (41.8)
LCX, *n* (%)	98 (21.4)
RCA, *n* (%)	153 (33.5)
ISR, *n* (%)	62/341 (18.2)
Average ISR time (month)	32.8 ± 26.8
ISR rate for vessel	
LM, *n* (%)	1/15 (6.7)
LAD, *n* (%)	41/191 (21.5)
LCX, *n* (%)	19/98 (19.4)
RCA, *n* (%)	22/153 (14.4)
Medication	
Statin therapy, *n* (%)	321/341 (94.1)
Antiplatelet therapy, *n* (%)	322/341 (94.4)
Clopidogrel	136 (39.8)
Ticagrelor	172 (50.4)
Aspirin	285 (83.5)
Oral anticoagulation, *n* (%)	1/341 (0.3)
ACEI/ARB, *n* (%)	298/341 (87.3)

*LM* left main coronary artery, *LAD* left anterior descending coronary artery, *LCX* left circumflex artery coronary artery, *RCA* right coronary artery, *ACEI/ARB* angiotensin-converting enzyme inhibitor/angiotensin receptor blocker

### Clinical and angiographic characteristics between patients with and without ISR

Clinical and angiographic characteristics of patients in ISR ( +) and ISR (−) group are presented in Table 2. There was no difference about the age and gender between ISR ( +) and ISR (−) group. Also, there was no difference for risk factors including smoking, drinking, hypertension, diabetes and dyslipidemia between the two groups. Patients in ISR ( +) group had significantly lower LVEF, antiplatelet therapy and ACE1/ARB treatment than those in ISR (−) group. As for angiographic characteristics, patients in ISR ( +) group had a high number of mean stents, and lower proportion of rapamycin eluting stents than those in ISR(-) group, but there was no difference for mean stent length and stent diameter between the two groups.

**Table 2:** Clinical and angiographic characteristics of patients with and without ISR

Variables	ISR ( +) group (*n* = 62)	ISR (−) group (*n* = 279)	*P* value
Age (years)	66.71 ± 9.65	65.63 ± 11.15	0.48
Male, *n* (%)	48 (77.4)	205 (73.5)	0.52
Smoking, *n* (%)	10 (16.1)	57 (20.4)	0.44
Drinking, *n* (%)	3 (4.8)	3 (1.1)	0.13
Hypertension, *n* (%)	37 (59.7)	178 (63.8)	0.54
Diabetes mellitus, *n* (%)	28 (45.2)	109 (39.1)	0.38
Dyslipidemia, *n* (%)	14 (22.6)	68 (24.4)	0.77
LVEF (%)	53.00 ± 15.47	56.72 ± 11.62	0.03
CRP (mg/L)	17.09 ± 33.67	42.03 ± 60.12	0.08
cTnI (nm/mL)	0.003 ± 0.194	0.003 ± 0.398	0.12
HbA1c (mm/L)	7.02 ± 1.90	6.99 ± 1.70	0.92
Follow-up (months)	32.8 ± 26.8	34.5 ± 14.3	0.48
Stent number	1.92 ± 0.87	1.21 ± 0.50	< 0.001
Stent length (mm)	25.32 ± 8.48	26.48 ± 7.48	0.06
Stent diameter (mm)	2.99 ± 0.60	2.98 ± 0.42	0.52
Stent type			
Rapamycin, *n* (%)	37 (59.7)	238 (84.9)	< 0.001
Other drug*, *n* (%)	25(40.3)	41(15.1)	
Medication			
Statin therapy, *n* (%)	51 (82.3)	270 (96.8)	< 0.001
Antiplatelet therapy, *n* (%)	53 (85.5)	269 (96.4)	< 0.001
Clopidogrel	23 (37.0)	113 (40.5)	0.795
Ticagrelor	21 (33.8)	151 (54.1)	0.004
Aspirin	41 (66.1)	244 (87.5)	< 0.001
ACEI/ARB, *n* (%)	50 (80.6)	248 (88.9)	0.001

*ISR* in-stent restenosis, *LVEF* left ventricular ejection fraction, *CRP* C reaction protein, *ACEI/ARB* angiotensin-converting enzyme inhibitor/angiotensin receptor blocker

^*^Other drug include Everolimus, Zotamos

### Comparison of serum lipid levels in ISR ( +) and ISR (−) group

Previous study suggested that lipid profiles were associated with coronary lesions after PCI [23], so we compared the follow-up serum levels of TC, TG, HDL-C, LDL-C, ApoA1 and ApoB in patients in ISR ( +) and ISR (−) group. As shown in Table 3, compared to ISR (−) group, patients in ISR ( +) presented significantly lower levels of serum TC, LDL and ApoB. The levels of serum HDL and ApoA1 were no different between the two groups.

**Table 3:** Serum lipid profiles in patients with ISR and non-ISR

Variables	ISR ( +) group (*n* = 62)	ISR (−) group (*n* = 279)	*P* value
TC (mmol/L)	4.60 ± 1.40	5.13 ± 1.37	< 0.01
TG (mmol/L)	2.48 ± 4.62	2.02 ± 1.47	0.17
HDL (mmol/L)	1.17 ± 0.30	1.15 ± 0.26	0.56
LDL (mmol/L)	2.54 ± 0.79	3.04 ± 1.01	< 0.01
ApoA1 (mmol/L)	1.03 ± 0.26	1.05 ± 0.27	0.51
ApoB (mmol/L)	0.84 ± 0.28	0.98 ± 0.37	0.02

*TC* total cholesterol, *TG* triglyceride, *HDL* high-density lipoprotein, *LDL* low-density lipoprotein

### Independent risk factors associated with ISR

**Fig. 1:**
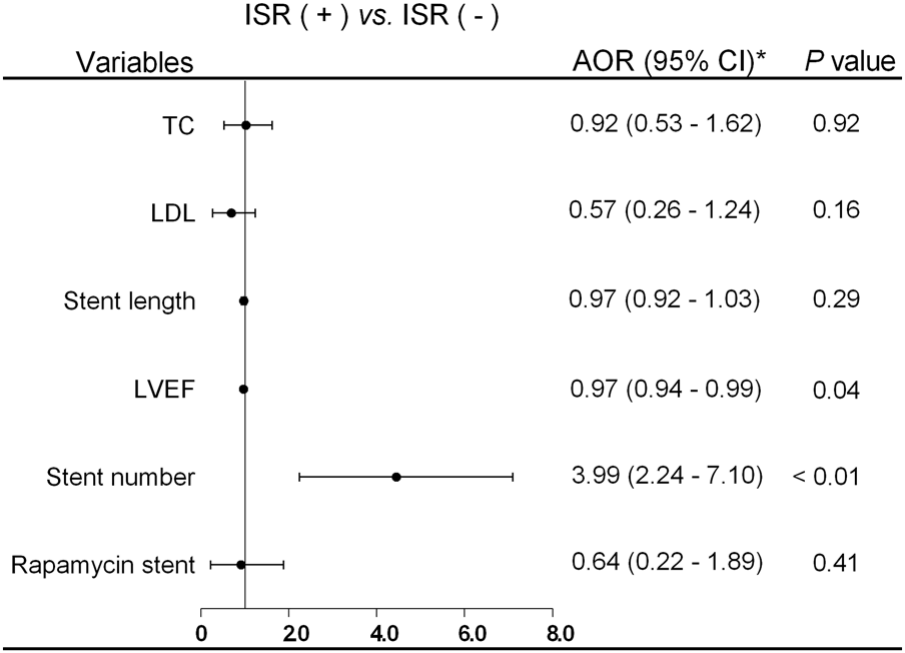
Multivariate logistic regression analysis of risk factors for ISR. *Adjusted for gender, age, hypertension, diabetes mellitus, statin therapy, ticagrelor therapy, aspirin therapy, ACEI/ARB therapy, and stent diameter. *OR* odd ratio, *CI* confidence interval, *TC* total cholesterol, *LDL* low-density lipoprotein, *LVEF* left ventricular ejection fraction

Multivariate logistic regression model was used to further evaluate the correlation between the variables and ISR. The data showed that TC, LDL, LVEF, stent number were significantly correlated with ISR (Table 4). After adjusting for the gender, age, hypertension, diabetes mellitus, statin therapy, ticagrelor therapy, aspirin therapy, ACEI/ARB therapy, and stent diameter, only stent number and LVEF were independent risk factors for ISR (Table 4 and Fig. 1).

**Table 4:** Logistic regression analysis of risk factors for ISR

Variables	Univariate		Multivariate*	
	OR (95%CI)	*P*	OR (95%CI)	*P*
TC (mmol/L)	0.76 (0.60–0.97)	0.03	0.92 (0.53–1.62)	0.92
LDL (mmol/L)	0.66 (0.47–0.91)	0.01	0.57 (0.26–1.24)	0.16
Stent length (mm)	0.96 (0.92–1.00)	0.07	0.97 (0.92–1.03)	0.29
LVEF (%)	0.98 (0.96–0.99)	0.03	0.97 (0.94–0.99)	0.04
Stent number	4.15 (2.73–6.31)	< 0.01	3.99 (2.24–7.10)	< 0.01
Rapamycin stent	0.57 (0.25–1.29)	0.176	0.64 (0.22–1.89)	0.41

*OR* odd ratio, *CI* confidence interval, *TC* total cholesterol, *LDL* low-density lipoprotein, *LVEF* left ventricular ejection fraction

^*^Adjusted for gender, age, hypertension, diabetes mellitus, statin therapy, ticagrelor therapy, aspirin therapy, ACEI/ARB therapy, and stent diameter

## Discussion

In this study, we reported the incidence and risk factors of ISR in ACS patients who underwent successful PCI with DES during a follow-up of approximately 2 years. Our data showed that the incidence of ISR was 18.2%. Stent number and LVEF were strongly associated with ISR. As most of the current knowledge of ISR was obtained from BMS stents, our findings may improve the understanding of relationship between clinical characteristics and ISR with DES, which would be practical help for the management and prevention of it.

PCI is the primary and effective tactics for treating CAD. However, although interventional approaches and pharmacological therapies have improved, ISR remains a major challenge in the stent era [24]. Patients with ISR need repeat revascularization which would increase mortality and affect patients’ life quality physically and psychologically [11]. Currently, although some percutaneous strategies are used to deal with ISR, there is still not standard treatment for it. Therefore, long-term secondary prevention is very important to reduce the occurrence of ISR in patients after PCI with stents. In the present study, we retrospectively reviewed patients who had been implanted with drug-eluting stents and were followed-up for coronary angiography. The clinical characteristics and angiographic factors were compared between patients with ISR and without ISR. Inflammation is known to play an essential role in coronary atherosclerosis process and cardiovascular events. As a marker of inflammation, the predictive value of CRP for clinical and angiographic outcome after PCI was conflicting [25, 26]. A recent study by Xu et al. showed that ISR rate was significantly higher in patients with CRP > 2 mg/l, and suggested that CRP might be of great value for prediction of ISR [23]. However, we found that CRP levels varied greatly in patients with ISR ( +) and ISR (−), and no significant difference was observed between these groups.

Dyslipidemia is a well-known risk factor of atherosclerosis, and but the association between serum levels and ISR remained less clear. Zairis et al. found that higher levels of HDL-C reduced ISR incidence and major adverse cardiac events [27]. Another study by Kim interestingly found that increased LDL-C particle size was associated with lower ISR rate [28]. However, another study by Xu et al. did not show an association between lipid profile and ISR. In the current study, to our surprise, the levels of TC and LDL were lower in patients in ISR (−) group, while no difference was observed for TG and HDL between two groups. This might in part be attributed to the higher dyslipidemia and higher proportion of statin therapy in ISR (−) group, and in part because of much smaller sample size in ISR ( +) groups. But after adjusted for other factors, multivariate logistic model showed no significant association between serum lipid profile and ISR.

The present study investigated the incidence of ISR in ACS patients after successful PCI during an angiographic follow-up of more than 2 years. The average time from stent implantation to the occurrence of ISR was 32.8 months, and ISR would tend to occur in LAD and LCX. Some studies have suggested that stent length an important determinant for ISR. Hong et al. found that stent length (> 40 mm) was an independent predictor of ISR development [29]. Observation from a study by Choi et al. indicated that patients treated with stents of length ≥ 32 mm had a greater risk of ISR than those treated with stents < 32 mm. Meanwhile, vessel diameter was correlated with ISR, as reported by the HORIZONS-AMI study, which found that vascular caliber ≤ 3 mm increased the incidence of ISR [30]. The present study compared the stent length and diameter between patients in ISR ( +) and ISR (−) groups, no significant difference was observed in these groups. However, the mean stent number of ISR ( +) group was significantly higher than that of ISR (−) group. Logistic model showed that stent number was a pronounced risk factor of ISR with an OR of 3.99. Previous studies have shown that patients with ISR presented lower levels of LVEF [31, 32]. Consistently, our study found that the LVEF of patients in ISR ( +) was significantly lower than those in ISR (−), and multivariate logistic model suggested an independent association between LVEF and ISR.

Our study have some limitations. First, patients received a second coronary angiography because of recurring symptoms. These patients tended to have inferior outcomes, which might lead to a higher ISR incidence than actual, and less different characteristics between ISR ( +) and ISR (−) groups. Second, this was retrospective design, and represented a single-center study with DES restenosis. Therefore, the results only suggested association between clinical factors and restenosis, but not the cause–effect relations.

## Conclusions

In conclusion, the present study investigated the ISR incidence of ACS patients after successful PCI with DES from southern China, and identified risk factors for ISR. Our data suggested that LVEF and stent number were independent risk factors associated with ISR.

## Data Availability

The datasets used and/or analyzed during the current study available from the corresponding author on reasonable request.

## Abbreviations

ACS: Acute coronary syndromes
CAD: Coronary artery disease
CIs: Confidence intervals
DBP: Diastolic blood pressure
DCB: Drug-coated balloon
DES: Drug-eluting stents
ISR: In-stent restenosis
LAD: Left anterior descending coronary artery
LCX: Left circumflex artery coronary artery
LM: Left main coronary artery
LVEF: Left ventricular ejection fraction
ORs: Odds ratios
PCI: Percutaneous coronary intervention
QCA: Quantitative coronary angiography
RCA: Right coronary artery
SBP: Systolic blood pressure
TC: Total cholesterol
TG: Triglyceride
